# Synthesis of nano-crystallite hydroxyapatites in different media and a comparative study for estimation of crystallite size using Scherrer method, Halder-Wagner method size-strain plot, and Williamson-Hall model

**DOI:** 10.1016/j.heliyon.2024.e25347

**Published:** 2024-01-30

**Authors:** Md. Kawsar, Md. Sahadat Hossain, Newaz Mohammed Bahadur, Samina Ahmed

**Affiliations:** a Glass Research Division, Institute of Glass & Ceramic Research and Testing, Bangladesh Council of Scientific and Industrial Research (BCSIR), Dhaka-1205, Bangladesh; b Department of Applied Chemistry and Chemical Engineering, Noakhali Science and Technology University, Noakhali, Bangladesh; c BCSIR Dhaka Laboratories, Bangladesh Council of Scientific and Industrial Research (BCSIR), Dhaka-1205, Bangladesh

**Keywords:** Crystallite size, X-ray diffraction, Peak profiling, Crystallographic parameter

## Abstract

Hydroxyapatite (HAp) [Ca_10_(PO_4_)_6_(OH)_2_] is remarkably similar to the hard tissue of the human body and the uses of this material in various fields in addition to the medical sector are increasing day by day. In this research, mustered oil, soybean oil, as well as coconut oil were employed as liquid media for synthesizing nanocrystalline HAp using a wet chemical precipitation approach. The X-ray diffraction (XRD) study verified the crystalline phase of the HAp in all the indicated media and discovered similarities with the standard database. Several prominent models such as the Scherrer's Method (SM), Halder-Wagner Method (HWM), linear straight-line method (LSLM), Williamson-Hall Method (W-M), Monshi Scherrer Method (MSM), Size-Strain Plot Method (SSPM), Sahadat-Scherrer Method (S–S) were applied for the determination of crystallite size. The stress, strain, and energy density were also computed from the above models. All the models, without the Linear straight-line technique of Scherrer's equation, resulted in an appropriate value of crystallite size for synthesized products. The calculated crystallite sizes were 6.5 nm for HAp in master oil using Halder-Wagner Method, and 143 nm for HAp in coconut oil using the Scherrer equation which were the lowest and the largest, respectively.

## Graphical abstract

**Figure:**
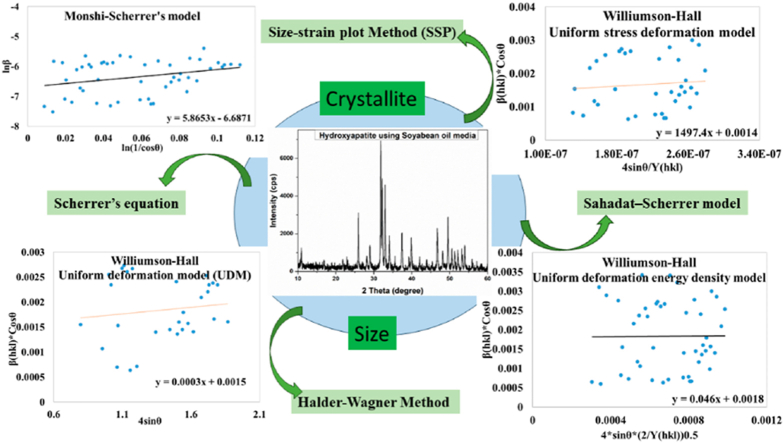

## 1 Introduction

The word ‘crystallite size,’ has a variation from ‘particle size,’ which is very much significant for crystalline substances for productive application [1]. And, crystallite size is particularly significant for microstructural and physical features of any crystalline materials [2]. Crystallographically, HAp has two distinct forms of structure: (a) monoclinic and (b) hexagonal. Hexagonal HAp contains space group of P63/m by a symmetrical axis of sixfold organized with a helix of threefold retaining a mirror plane and the intrinsic properties were reported as (a) crystal density = 3.140 g cm^−3^, (b) a = b = 9.42 Å and c = 6.88 Å, and (c) cell volume = 530.301 (Å)^3^ [3,4]. For the assessment of particle size along with crystallite size light scattering, BET (Brunauer–Emmett–Teller) theory, transmission electron microscopy (TEM), Atomic force microscopy (AFM), and scanning electron microscopy (SEM) are most often utilized [5,6]. A few other less applicable techniques are selected area electron diffraction (SAED), electron back-scattered diffraction (EBSD), and neutron diffraction (ND) [7]. The technique of X-ray diffraction (XRD) is acknowledged as an efficient and resilient instrument for determining the size of crystals [8,9]. Mainly, the Powder X-ray diffraction (PXRD) assessment method is widely utilized to evaluate the quantity of crystallite and deformation of the lattice. The knowledge of the quantity of crystallite and deformation of the lattice is associated with the diffraction peak extending inducing lattice strain derived from the researched material's defects (stacking defects or coherency stresses) [10].

Scientists generally utilize the traditional Scherrer equation to compute the crystallite size, which was introduced in 1918 [11]. However, until now, multiple modified formulas and models, e.g., the model of straight line passing through the origin (MSLPO) of the Scherrer equation, straight line model in Scherrer method (SLMSM), Williamson–Hall (W–H) model, Monshi–Scherrer model, Halder-Wagner (H–W) and Size-Strain Plot (SSP) methods have been established and utilized in many studies [[12], [13], [14], [15]]. The Scherer's approach is often used for determining crystallite size based on the broadening of the reflection. Nevertheless, the crystallite size estimated by this approach is a tiny bit erroneous since peak broadening develops with lattice strain and crystallite dimension [16,17]. The important component of SLMSM is that rather than selecting a specific reflection peak, it examines all peaks to determine the crystallite size. Furthermore, in the current works, it's shown that this strategy is erroneous in the scenario of natural nanocrystalline material [8]. In an additional technique, the Monshi–Scherrer equation was built employing the ln(log 10) edition of the Scherrer equation to compute crystallite size. Researchers have also pointed out this model to acquire more precise estimations for crystallite size as it keeps the relevance of lowering the deficiencies by leveraging the least squares approach and diminishing the absolute extent of limitation [1,13]. The Williamson–Hall developed an X-ray peak profile analysis approach which is employed to estimate the crystallite size D(hkl), stress (σ), lattice strain (ε), and energy density (u). The W–H technique is divided into three sub-methods Uniform deformation energy-density model (expressed as UDEDM), uniform stress deformation model (expressed as USDM), and uniform deformation model (expressed as UDM) to predict an idea of strain and stress-strain relation as a relation of energy density (u) [18]. Furthermore, when the crystals of the materials are isotropic, it is thought that the strain inside the crystal is uniform through all orientations and UDM is performed to quantify lattice strain [19,20]. If the sample comprises anisotropic crystals that are crystallographic planes composed of homogenous stress, the USDM can be leveraged for predicting anisotropic lattice stress [21]. Another model, UDEDM is also employed to estimate the crystallite size together with the energy density per unit of volume inside of crystals as a whole and is reliant on Hooke's law [22]. Additionally, SSP interprets the size-widened component as the function of the Lorentzian structure and the Gaussian function for the strain-broadened section of the XRD pattern. The benefit of the SSP approach is to pay extra significance to the lower-angle XRD diffraction peak, whereby the reliability and accuracy of the XRD data are great. For this reason, crystallite size computation from the SSP model is reported as more specific than the W–H technique [[23], [24], [25]]. Conversely, the H–W Method views the peak widening as a voigt function and presumes that typical strain and size may be deduced from the XRD peak widening [26]. In this work, we intend to find the crystal size of synthesized HAp (in water and oil media) using the aforementioned XRD models and analyze their elastic behavior.

## 2 Materials and methods

### 2.1 Materials

Orthophosphoric acid (H_3_PO_4_), ammonium hydroxide (NH_4_OH), calcium hydroxide (Ca(OH)_2_), and nitric acid (HNO_3_) were bought from E-Merck Germany. The substances employed in this research were analytical grade. The liquid organic media (Mustard oil, Soybean oil, Coconut oil) are acquired from the local market. A double distillation method was used to prepare the deionized (DI) water.

### 2.2 Synthesis method of nanocrystalline HAp

The study focuses on the use of mustard oil, soybean oil, and coconut oil as liquid media for synthesizing nano-crystalline hydroxyapatite (HAp) using a wet chemical precipitation method. These oils are natural, renewable, and biodegradable, making them environmentally friendly [[27], [28], [29], [30]]. They can also act as surfactants or structure-directing agents, influencing the growth and nucleation of HAp nanoparticles [27,30]. Additionally, their chemical compositions and molecular structures can influence the nucleation and growth of hydroxyapatite nanoparticles, resulting in variations in the crystalline structure and properties of the synthesized HAp.

Initially, an equal volume of 1.67 M Ca(OH)_2_ suspension and 1.0 M H_3_PO_4_ solution was prepared for the synthesis of HAp in water solvent. The ratio of the organic medium (Mustard oil, Soybean oil, Coconut oil) and the water mixture was maintained at 50:50 (vol%). H_3_PO_4_ was dropwise added to the calcium hydroxide suspension maintaining rate of 1 mLmin^-1^ fixing reaction parameters such as (a) 10–11 of solution pH (maintained by adding dilute ammonia solution and/or nitric acid); and (b) reaction temperature: at room temperature (25 °C). A vigorous stirring (300 rpm) was applied to produce the reaction. Subsequently, a precipitate was produced that was sorted out and then oven-dried maintaining 105 °C for 6 h. The entire dry component was crushed to powder and exposed to sintering at 900 °C for 0.5 h (increment steps were 3.5 °C min^−1^).

### 2.3 X-ray crystallographic characterization

Phase analysis of the synthesized HAp was accomplished utilizing an X-ray diffractometer (Model: Rigaku SE). With the measuring range at 2θ = 10°–60°, the findings were acquired in an ongoing scanning mode whilst the scanning steps were 0.01. The X-ray radiation source CuKα (λ = 1.54060 Å) was operated under conditions of 40 mA current and 50 kV voltage, while the chiller temperature was fixed at 22–23 °C. A similar experiment was taken out employing all the HAp samples derived from mustard oil, soybean oil, and coconut oil along with calcining at 900 °C. All the discovered reflected peaks were recognized by comparing them with the standard ICDD database files. Before evaluating the manufactured hydroxyapatite, the equipment was calibrated using a standard silicon reference sample which was also used to anticipate the instrumental broadening.

## 3 Results and discussion

### 3.1 XRD data analysis

**Fig. 1:**
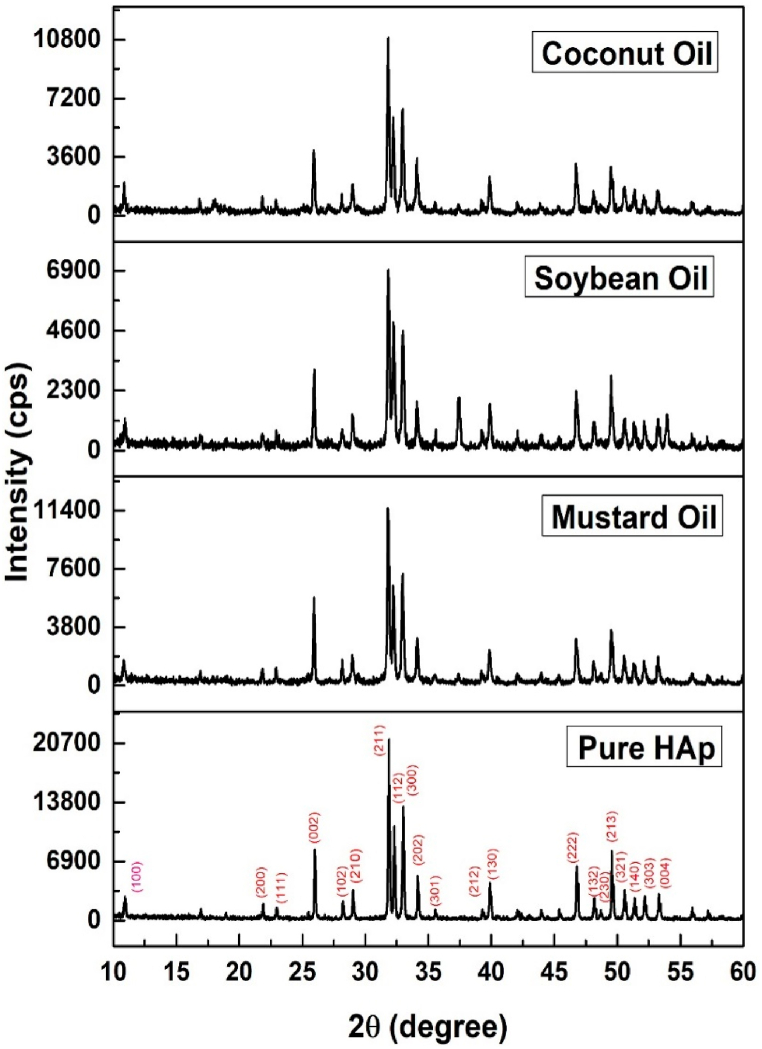
X-ray diffractogram of calcined hydroxyapatites using different organic media.

The XRD patterns of the synthesized HAp employing organic media such as mustard oil, soybean oil, as well as coconut oil are exhibited in Fig. 1, and the crystallographic parameters were examined from the developed patterns. The 2θ (degree) diffracted positions of the HAp phases were visualized at 25.93 (002), 31.83 (211), 32.24 (112), 32.96 (300), 34.12 (202), 39.88 (130), 46.75 (222), and 49.53 (213), which were matched with the standard ICDD database of the card no: #01-074-0565 for hydroxyapatite and a hexagonal structure was predicted. A very similar form of data was observed for all the synthesized HAp.

The crystallographic study assesses crystalline features such as lattice parameters, cell volume, crystallinity index, dislocation density, crystallite size, degree of crystallinity, and microstrain utilizing equations (1), (2), (3), (4), (5), (6), (7)), as mentioned as well as explained elsewhere [[31], [32], [33]].

(1) $$\text{Lattice}\;\text{parameter}\;\text{equation},{\left(\frac{1}{d_{\text{hkl}}}\right)}^{2}=\frac{4}{3}\left(\frac{h^{2}+\text{hk}+k^{2}}{a^{2}}\right)+\frac{l^{2}}{c^{2}}$$

(2) $$\text{Cell}\;\text{volume},V=\frac{\sqrt{3}}{2}a^{2}c$$

(3) $$\text{Crystallite}\;\text{size},D_{c}=\frac{K\lambda }{\beta \;\cos\;\theta }$$

(4) Microstrain,ε=β/4tanθ

(5) $$\text{Degree}\;\text{of}\;\text{crystallinity},X_{c}={\left(\frac{K_{a}}{\beta }\right)}^{3}={\left(\frac{0.24}{\beta }\right)}^{3}$$

(6) $$\text{Dislocation}\;\text{density},\delta =\frac{1}{{\left(D_{c}\right)}^{2}}$$

(7) $$\text{Crystallinity}\;\text{index},{\text{CI}}_{\text{XRD}\;}=\sum \frac{H_{\left(202\right)}+H_{\left(300\right)}+H_{\left(112\right)}}{H_{\left(211\right)}}$$

In the aforementioned equations, the unit cell is denoted by plane (h,k,l) and a,b,c represents lattice parameters, Dc = size of repeating unit, β = FWHM (full width at half maximum) in radian; θ = angle of diffraction (in degree), Xc = crystallinity degree, K = shape factor (arbitrary constant)/Scherrer's constant = 0.94 [34], δ = dislocation density, H_(*hkl*)_ = peak height of the respective plane, Ka = 0.24, for HAp, and CI_XRD_ = crystallinity index. By utilizing eq^n^ (8), the specific surface area of the synthesized HAp was calculated, where crystallite size and density of HAp were represented by Dc and ρ (3.16 g cm^−3^) [35].

(8) $$\text{Specific}\;\text{surface}\;\text{area},s=\frac{6}{\rho \times D_{c}\;}\;g^{‐1}m^{2}$$

Crystallite sizes in ordered materials are significant in diverse applications, since small crystallites are characterized by large surface areas, and vice versa [36].

The physical arrangement of atoms or molecules in any well-aligned material in a three-dimensional frame is revealed by the degree of crystallinity. The crystallinity level considerably determines the features of materials, however properly managing crystallinity is quite challenging. From the study, it's evident that HAp demonstrates diverse magnitudes in the degree of crystallinity. The data was calculated using equation (5).

In crystalline solids, microstrain corresponds to the intrinsic stress of crystal planes, which might emerge as either compressive or tensile forces. As a consequence of microstrain, crystallite deformation occurs, giving conception to changes in the properties of substances, notably in suitability. A constant variation in microstrain has emerged from the estimated data produced by applying equation (4).

Imperfection in crystalline materials is displayed because of several flaws such as point dislocation, line dislocation, and area dislocation, frequently known as dislocation. Dislocation density analyzes the number of dislocation lines per given surface area and is directly linked to the crystal size [37]. However, the amount of line dislocation is computed using equation (6). The data is registered in Table 1. Crystallite sizes in ordered materials are significant in diverse applications, since small crystallites are characterized by large surface areas, and vice versa. The crystallinity index (CI_XRD_) is discussed for assessing the numerical quantification of crystal structure. In this specific section, the X-ray diffraction (XRD) data were evaluated to compute the crystallinity using equation (7), as well as the results obtained are displayed in Table 1.

**Table 1:** Crystallographic parameters of the prepared HAp samples by using different organic media.

Parameter	Pure HAp	Mustard oil	Soybean oil	Coconut oil
Lattice parameter	a = b = 9.39 c = 6.82	a = b = 9.40 c = 6.87	a = b = 9.40 c = 6.872	a = b = 9.41 c = 6.87
Crystallite size, nm	81.97	65.75	51.88	54.93
Degree of crystallinity	13.41	6.96	3.40	4.063
Microstrain, ε	1.54 × 10^−3^	1.923 × 10^−3^	2.4407 × 10^−3^	2.30 × 10^−3^
Dislocation density, (10^15^ lines per m^2^)	0.1488	0.2313	0.3715	0.3313
Crystallinity index, CI_XRD_	1.46	1.31	1.52	1.48
Specific surface area, S (g^−1^m^2^)	0.02316	0.02887	0.03659	0.03456
Volume of unit cell(Å^3^)	521.416	526.188	525.859	527.598

### 3.2 Crystallite size calculation using various models

Precise crystallite size estimation for each application is a crucial criterion. Yet, for determining the dimension of the crystallite of HAp samples, multiple approaches and mathematical equations have been used including Scherrer's Method (SM), linear straight-line method (LSLM), Size-Strain Plot Method (SSPM), Williamson-Hall Method (WHM), Monshi Scherrer Method (MSM), Sahadat-Scherrer Method (S–S), Halder-Wagner Method (HWM). The Williamson-Hall Method was further diversified emphasizing the UDSM, UDM, and UDEDM models.

#### 3.2.1 Scherrer's method (SM)

Scherer's formula for ideal conditions for diffraction on monodisperse particles of crystalline materials with homogeneous organized domains was developed for parallel, monochromatic, and infinitely thin X-ray beams [38]. The broadening of XRD spectra in nanocrystals is linked to non-intrinsic strain effects and crystallite size, involving instrumental and physical expanding components [15]. To minimize this inaccuracy of the instrument, equation (9) may be deployed:

(9) $$\beta_{d}=\sqrt{\beta_{m}^{2}-\beta_{i}^{2}}$$

In equation (9), β_m_ is the determined broadening, β_i_ is the broadening by the instrumental, and β_d_ is established as the modified broadened accountable for crystal dimension. However, the physical and instrumental broadening of the sample was measured through the full-width at half maxima (FWHM). So, we could calculate the average crystal size and overlook the influence of the strain by using the Scherrer method with the following equation (10) [39].

(10) $$\text{Crystallite}\;\text{size},D_{c}=\frac{K\lambda }{\beta \text{Cos}\;\theta }$$

Here, D_c_ indicates the crystallite size, K is the shape constant (K is equal to 0.9, for cubic crystal), wavelength (λ) of the Cu-radiation was 1.54056 Å for CuKα_1_ radiation, β is the full width of the reflection at half of the maximum intensity, and the diffraction angle is θ. The crystal sizes obtained from this model were 83.23 nm for Pure HAp, 119.33 nm for Mustard oil, 105.68 nm for Soybean oil, and 143.41 nm for Coconut oil.

#### 3.2.2 Liner straight-line method of Scherrer's equation (LSLMSE)

**Fig. 2:**
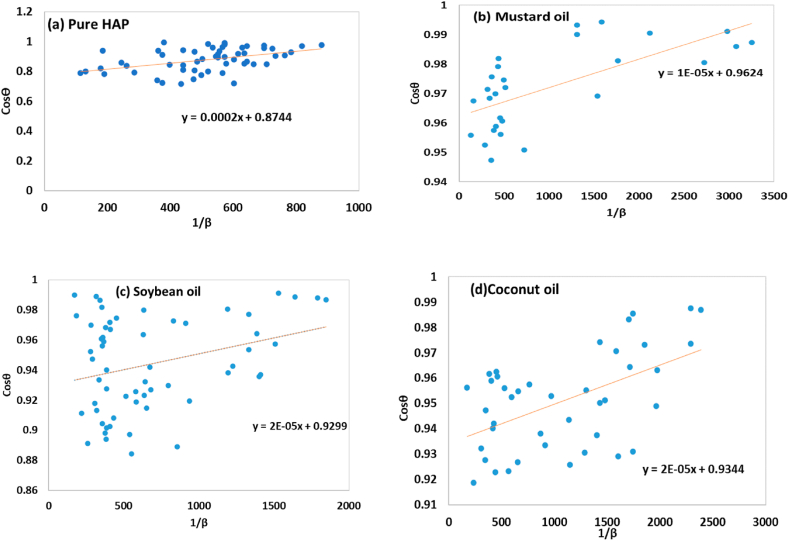
Determination of crystallite size using liner straight line model of Scherrer equation for (a) Pure HAp (b) Mustard oil (c) Soybean oil and (d) Coconut oil.

The fundamental requirement of the LSLMSE method was to evaluate all peaks in synthesized samples (shown in Fig. 2(a–d)), rather than focusing on a specific scattering peak [8]. The Scherrer equation can be reformed in the new equation (11). This resultant equation can be used to estimate crystallite size (D_L_), widely articulated in various studies [39,40]. The mathematical Equation for this model can be written as follows:

(11) $$\mathrm{Cos}\;\theta =\frac{K\lambda }{\text{Dc}}\times \frac{1}{\beta }=\frac{K\lambda }{D_{L}\;}\times \frac{1}{\beta }$$

The crystallite size, denoted by DL, was calculated using the LSLMSE method. A graph was constructed by plotting Cosθ as well as 1/β on the Y-axis and X-axis respectively. The crystal size was measured using the gradient m = Kλ/(DL), were 2 × 10^−4^, 1 × 10^−5^, 2 × 10^−5^, and 2 × 10^−5^ were obtained for pure HAp, mustard oil, soybean oil, and coconut oil correspondingly. The estimated crystal size was found to be 693.27 nm for pure HAp, 13865.4 nm for mustard oil, 6932.7 nm for soybean oil, and 6932.7 nm for coconut oil.

#### 3.2.3 Monshi–Scherrer method (MSM)

The Scherrer equation reveals higher nanocrystalline size when d-spacing as well as 2θ values drop. Modifying the equation may eliminate shortcomings or Σ(±Δln β)^2^ to offer a more accurate evaluation of crystal size from all or a part of unique peaks, enhancing the stability of β. Cosθ [13]. The Monshi–Scherrer model is shown in equation (12), with the dimension of the crystallite being indicated by D_M–S_ [41].

(12) $$\ln\;\beta =\ln\;\frac{1}{\text{Cos}\theta }+\ln\frac{K\lambda \;}{D_{M-s}}$$

**Fig. 3:**
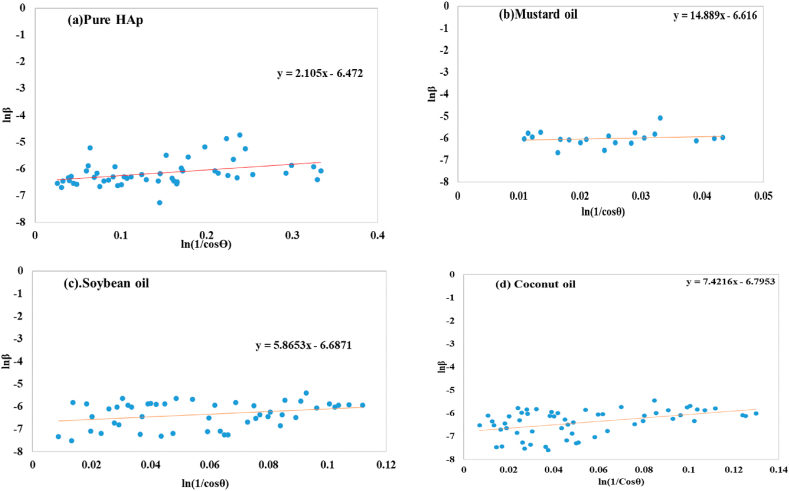
Determination of crystallite size using Monshi–Scherrer method equation for (a) pure HAp (b) mustard oil (c) soybean oil (d) coconut oil.

This model was examined by plotting lnβ and ln 1/cosθ on the Y-axis, and X-axis (visualized in Fig. 3(a–d)). The straight-line equation (y = mx + c) and equation (12) were compared with each other to find out the slope, which gives a checkpoint for verifying the correctness of the findings.

The plot showed that the values of slope as 2.105 for pure HAp, 14.889 for mustard oil, 5.8653 for soybean oil, and 7.4216 for coconut oil. The crystal size values for pure HAp, mustard oil, soybean oil, and coconut oil were computed, with a total of 89.67, 103.57, 111.19, and 123.91 nm, correspondingly.

#### 3.2.4 Williamson–Hall method (WHM)

The modified Scherrer's Equation addresses crystallite size impact through XRD reflection, neglecting inherent strain in nanocrystals. However, intrinsic strain is crucial due to grain boundaries, point defects, dislocations, and stacking in nanocrystals [42]. Consequently, the Williamson-Hall approach analyzes strain from XRD peak widening, following estimating intrinsic strain from crystallite size values. This approach is more accurate for calculating crystallite size with several reflected peaks [43]. Eventually, the overall broadening may be described as equation (13) [15].

(13) $$\beta_{Total}=\beta_{size}+\beta_{strain}$$

where β_size_ is the broadening due to its size and β_strain_ is connected to the strain broadening effect. The modified form of the Williamson-Hall considers a UDM, USDM, UDEDM, and the sizes-strain plot method (SSP) will be discussed in this context [44].

##### 3.2.4.1 Uniform deformation model (UDM)

The estimation of strain obtained by crystalline defects and distortion in the synthesized powder can be mathematically expressed as equation (14) [45]:

(14) $$\epsilon =\frac{\beta_{\text{hkl}}}{4\;\tan\;\theta }$$

The UDM is founded on the concept that the strain is considered uniform in all orientations. The lattice strain is consequently perceived as an isotropic property that is independent of the extent of direction [46]. The peak broadening occurred by lattice strain can be presented as equation (15):

(15) $$\beta_{\text{strain}\;}=4\epsilon \;\tan\;\theta$$

The overall broadening, β_hkl_ reflecting the FWHM of a reflected peak which is related to the influence of the strain of crystal lattice (β_strain_) and the value of the size of the crystals (β_size_) in a specific peak that may be stated as equations (16), (17), (18).

(16) $$\beta_{\;\text{hkl}}=\beta_{\text{size}}+\beta_{\text{strain}}$$

(17) $$\beta_{\text{hkl}=}\;\frac{K_{\beta }\lambda }{D_{W}.\text{cos}\theta }+4\epsilon \;\tan\;\theta$$

Equation (9) can be written as:

(18) $$\beta_{\text{hkl}}c\text{os}\theta =\frac{K_{\beta }\lambda }{D_{W}}+4\epsilon s\text{in}\theta$$

**Fig. 4:**
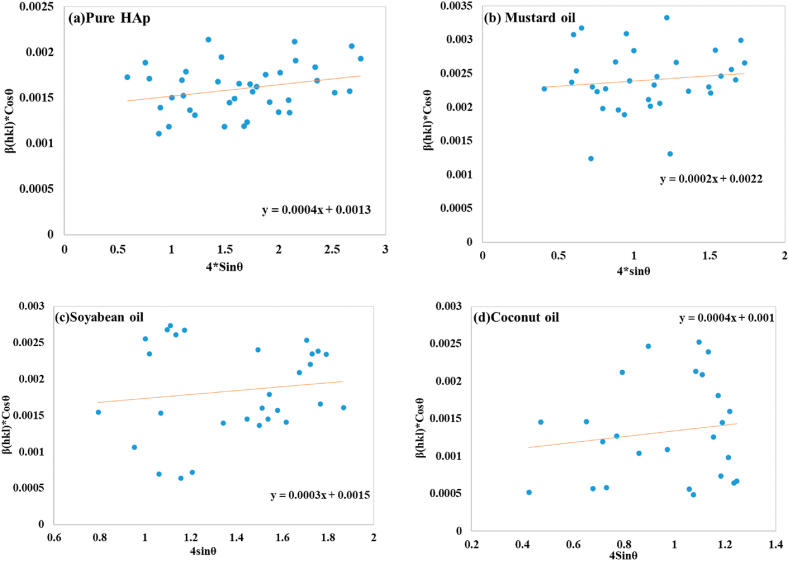
Determination of crystallite size using Uniform deformation model for (a) Pure HAp (b) Mustard oil (c) Soybean oil (d) Coconut oil.

The linear equation, incorporating 4sinθ (X-axis) and β_hkl_*cosθ (Y-axis), enables for the estimation of slope (ε) and crystallite size (D_w_) in a graph, as seen in Fig. 4(a–d), and given in Table 2.

**Table 2:** Microstructural characteristics of hydroxyapatite utilizing various models in this study.

Model Name		Crystal size (in nm), strain (in N/m^2^), energy density (in J/m^3^)			
		Pure HAp	Mustard Oil	Soybean Oil	Coconut oil
Scherrer's Model		83.23	119.33	105.68	143.41
The linear straight-line method of Scherrer's equation		693.27	13865.4	6932.7	6932.7
Monshi- Scherrer's Method		89.68	103.57	111.19	123.91
Williamson-Hall Method	UDM	ε= 0.0004 D_w_ = 106.66	ε =0.0002 D_w_ = 63.02	ε = 0.0003 D_w_ = 92.44	ε =0.0004 D_w_ = 138.65
	USDM	σ = 1015.2 D_(*hkl*)=_ 99.04	σ = 1877.9 D_(*hkl*)=_ 99.04	σ = 1497.4 D_(*hkl*)=_ 99.04	σ = 414.66 D_(*hkl*)=_ 99.04
	UDEDM	u = 4.52 × 10^−3^ Dw = 98.32	u = 1 × 10^−14^ Dw = 77.03	u = 2.116 × 10^−3^ Dw = 77.0	u = 70.11 × 10^−3^ Dw = 99.04
Size-strain plot		53.33	38.52	37.45	26.16
Halder-Wagner Method		47.61	6.5	43.48	34.48
Sahadat-Scherrer's Model		86.66	106.66	126.05	138.65

The slope of the UDM curve reveals the existence of intrinsic strain, a phenomenon involving the lattice expansion of nanocrystals [47]. Pure HAp as well as coconut oil had a slope of 4 × 10^−4^, whereas mustard oil and soybean oil had slopes of 2 × 10^−4^ and 3 × 10^−4^ respectively. The estimated crystal size of pure HAp was 106.66 nm, whereas mustard oil had 63.02 nm, soybean oil had 92.44 nm, and coconut oil had 138.65 nm.

##### 3.2.4.2 Uniform stress deformation model (USDM)

The Uniform Stress Deformation Model (USDM) was validated by incorporating the anisotropic character into lattice strain analysis. This modified model focuses on the lattice deformation stress for crystal plane directions with low microstrains uniformly, addressing the issue of sample uniformity and the potential anisotropic nature of actual crystals [42]. Hooke's law, expressed as equation (19), relates stress (σ) and strain (ε), with higher accuracy for low-stress values [48].

(19) $$\sigma =Y_{\text{hkl}}\varepsilon$$

where Y_hkl_ is Young's modulus or modulus of elasticity, the mathematical expression is just an approximation that is reliable for a minimal strain. Furthermore, raising the strain produces a variation of particles from being linear in nature, respectively [49]. In this context, 6 × 10^9^ N/m^2^ is considered the value for Young's modulus [50]. By rearranging and replacing equation (19) with equation (18), we have the following relation (equation (20)):

(20) $$\beta_{\text{hkl}}\text{cos}\theta =\frac{K_{B}\lambda }{D_{\text{hkl}}\;}+4\;\sigma \frac{\;\text{sin}\theta }{Y_{\text{hkl}}}$$

**Fig. 5:**
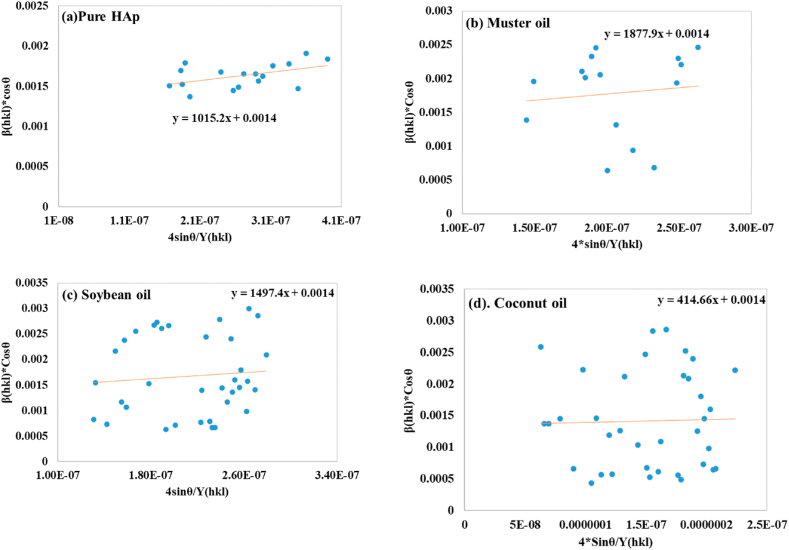
Determination of crystallite size using Uniform stress deformation model for (a) pure HAp (b) mustard oil (c) soybean oil (d) coconut oil.

A linear graph was generated by plotting β_total_*cosθ and 4*Sinθ/Y_(*hkl*)_ along the Y-axis as well as X-axis. The gradient of this straight-line measured stress (σ) and crystallite size D_(*hkl*)_ of HAp nanocrystals. The crystal size was estimated from the uniform stress deformation model, with 99.04 nm for pure HAp, 99.04 nm for mustard oil, 99.04 nm for soybean oil, and 99.04 nm for coconut oil. The stress calculated was 1015.2 (N/m^2^) for pure HAp, 1877.9 (N/m^2^) for mustard oil, 1497.4 (N/m^2^) for soybean oil, and 414.66 (N/m^2^) for coconut oil. The plots are illustrated in Fig. 5(a–d), and the computed σ and D_(*hkl*)_ values are registered in Table 2.

##### 3.2.4.3 Uniform deformation energy density model (UDEDM)

The orientational arrangement in UDM necessitates adjustment of the W–H relationship from anisotropic crystal, since isotropy and homogeneity are not essential for efficient arrangement [51]. The relationship between stress (σ) and strain (ε) in USDM is linear, based on Hooke's law. However, in actual crystalline materials, defects like agglomerations and dislocations cause imperfections. UDEDM examines crystal imperfection, anisotropic deformation, and distortion as expressions of energy density (u), ensuring that constants related to stress and strain remain independent [52]. The u (energy per unit volume) as an expression of ε is determined by Hooke's expression as equation (21).

(21) $$u=\varepsilon^{2}\;\frac{Y_{\text{hkl}}}{2}$$

The UDEDM equation can be obtained by reorganizing equation (13) to ε and replacing it with equation (9), resulting in equation (22).

(22) $$\beta_{\text{hkl}}\text{Cos}\theta =\frac{K_{B}\lambda }{D_{w}\;}+4\text{Sin}\theta \surd u\frac{\surd 2}{\surd Y_{\text{hkl}}}$$

**Fig. 6:**
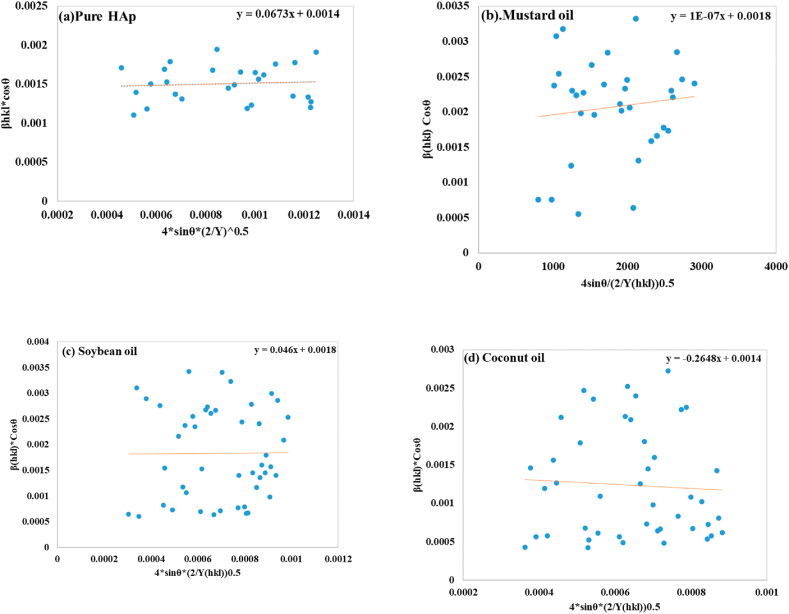
Determination of crystallite size using Uniform deformation energy density model for (a) pure HAp (b) mustard oil (c) soybean oil (d) coconut oil.

The graph plotted (shown in Fig. 6(a–d)), between $\beta_{\left(\text{hkl}\right)}\;\cos\;\theta$ (Y-axis) as well as $4\text{Si}n\theta \;\frac{\surd 2}{\surd Y_{\text{hkl}}}$ (X-axis) to estimate anisotropic energy density (u) and crystallite size (Dw). The energy density was reported as 4.52929 × 10^−3^ (J/m^3^) for pure HAp, 1 × 10^−14^ (J/m^3^) for mustard oil, 2.116 × 10^−3^ (J/m^3^) for soybean oil, and 70.11 × 10^−3^ (J/m^3^) for coconut oil, whereas Crystal size was measured 98.32 nm for pure HAp, 77.03 nm for mustard oil, 77.03 nm for soybean oil, and 99.04 nm for coconut oil, correspondingly.

#### 3.2.5 Size-strain plot method (SSP)

The Size-strain plot is a method used to determine XRD peak broadening, incorporating both Gaussian as well as Lorentz functions. The widening is regulated by strain, with the crystallite size of synthesized materials contributing to the broadening, indicated as a Lorentz function [42]. This model employs data at decreased diffraction angles to compensate for the lack of accuracy and significant peak overlaps caused to higher-degree influence planes [53]. So, the total SSP peak widening may thus be represented as the summation of broadening generated by Lorentzian (β_L_) and Gaussian (β_G_) functions (equation (23)).

(23) $$\beta_{\text{total}}=\beta_{L}+\beta_{G}$$

The SSP is mathematically expressed using equation (24) [54,55].

(24) $${\left(d_{\text{hkl}}\beta_{\text{hkl}}\text{cos}\theta \right)}^{2}=\frac{K_{B}\lambda }{D_{w}\;}\;\left({d_{\text{hkl}}}^{2}\beta_{\text{hkl}}\text{cos}\theta \right)+\frac{\varepsilon^{2}}{4}$$

where d_hkl_ is the lattice distance between the (hkl) planes or d-spacing. Which was measured by the following equation (25).

(25) $$d_{\text{hkl}}=\sqrt{\frac{a^{2}}{h^{2}+k^{2}+l^{2}}}$$

**Fig. 7:**
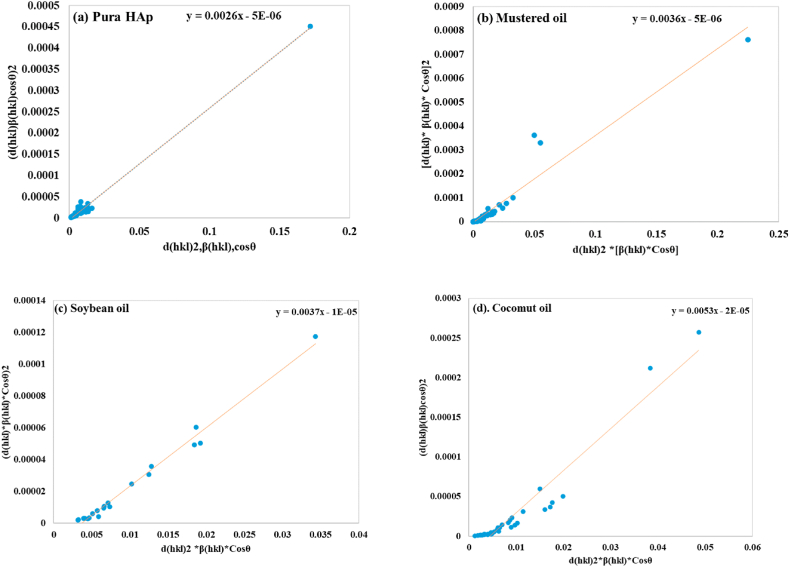
Determination of crystallite size using Stress-Strain plot for (a) Pure HAp (b) Mustard oil (c) Soybean oil (d) Coconut oil.

A linear fit is produced by plotting the term $\left({d_{\text{hkl}}}^{2}\beta_{\text{hkl}}\;\cos\;\theta \right)$ on the X-axis and ${\left(d_{\text{hkl}}\beta_{\text{hkl}}\;\cos\;\theta \right)}^{2}$ on the Y-axis as a function of all XRD reflections (Fig. 7(a–d)). The linear fit slope and Y-intercept determine the average size of crystallite (Dw) and intrinsic strain (ϵ). The slop values equate to $\frac{K_{B}\lambda }{D_{w}\;}$, with values given as 0.0026 for pure HAp, 0.0036 for mustard oil, 0.0037 for soybean oil, and 0.0053 for coconut oil. The intercept causes the intrinsic strain, with a value equal to $\frac{\varepsilon^{2}}{4}$ , making the computation of intrinsic strain impossible for the synthesized samples. The crystal sizes found from this model are 53.33 nm for pure HAp, 38.515 nm for mustard oil, 37.47 nm for soybean oil, and 26.16 nm for coconut oil.

#### 3.2.6 Halder-Wagner method (HWM)

The SSP approach employs the tension expansion as the Gaussian function and size widening as the Lorentzian function in the XRD pattern. However, the XRD peak area matches the Gaussian function, with significant tail collapse. The bottoms of the profile fit the Lorentz function, but not the XRD peak [56,57]. That is why the Halder-Wagner technique approach demands the symmetrical Voigt function, a convolution that integrates Lorentzian and Gaussian functions [57,58]. Thus, the Voigt function may be expressed as equation (26), which represents the FWHM of the physical profile.

(26) $${\beta_{\text{hkl}}}^{2}=\beta_{L}\;\beta_{\text{hkl}}+{\beta_{G}}^{2}$$

Where, $\beta_{L}$ = FWHM for Lorentzian function. $\beta_{G}$ = FWHM for Gaussian function. This technique offers a higher weight to the Bragg peaks in the small and intermediate angle, and the overlapping of the reflection peaks was minimal, and the correlation between the crystallite size and the lattice ε relates to the H–W technique expressed by equations (27), (28), (29) [15].

(27) $${\left(\frac{{*\beta }_{\text{hkl}}}{*d_{\text{hkl}}}\right)}^{2}=\frac{1}{D_{w}\;}{\left(\frac{{*\beta }_{\text{hkl}}}{{{*d}_{\text{hkl}}}^{2}}\right)}^{.}+{\left(\frac{\varepsilon }{2}\right)}^{2}$$

(28) $$\text{Here},*{\beta_{\text{hkl}}}^{.}=\frac{\;\beta_{\text{hkl}\;}\;c\mathrm{os}\left(\theta \right)}{\lambda }$$

(29) $$*{d_{\text{hkl}}}^{.}=\frac{\;2d_{\text{hkl}\;}\;\sin\left(\theta \right)}{\lambda }$$

**Fig. 8:**
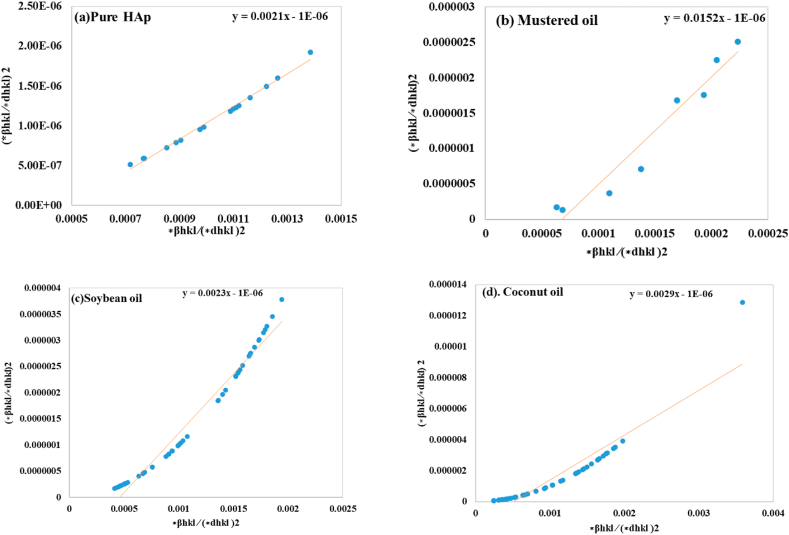
Determination of crystallite size using Halder- Wagner model for (a) Pure HAp (b) Mustard oil (c) Soybean oil (d) Coconut oil.

A plot of (∗ β_*hkl*_ ∕ ∗d_hkl_)^2^ on the Y-axis and∗ β_*hkl*_ ∕(∗d_hkl_)^2^ on X-axis yields a straight line (presented in Fig. 8(a–d)), with a slope equal to 1/D_w_, calculating microstrain. The values of the slope were reported as 0.0021 for pure HAp, 0.0152 for mustard oil, 0.0023 for soybean oil, and 0.0029 for coconut oil, corresponding crystallite diameters of 47.61, 6.57, 43.48, and 34.48 nm, respectively.

#### 3.2.7 Sahadat–Scherrer model

The Sahadat-Scherrer model offers an accurate approximation of the crystallite size, despite various limitations connected to previously stated models possibly leading to enormous crystallite sizes [1]. The technique involved studying each peak like previous models, but with a distinct linear route passing through the origin, resulting in a more precise model due to the straight line crossing through the origin [59]. The mathematical expression of this model can be written as equation (30):

(30) $$\text{Cos}\theta =\frac{K\lambda }{D_{S-S}}\times \frac{1}{\text{FWHM}}$$

**Fig. 9:**
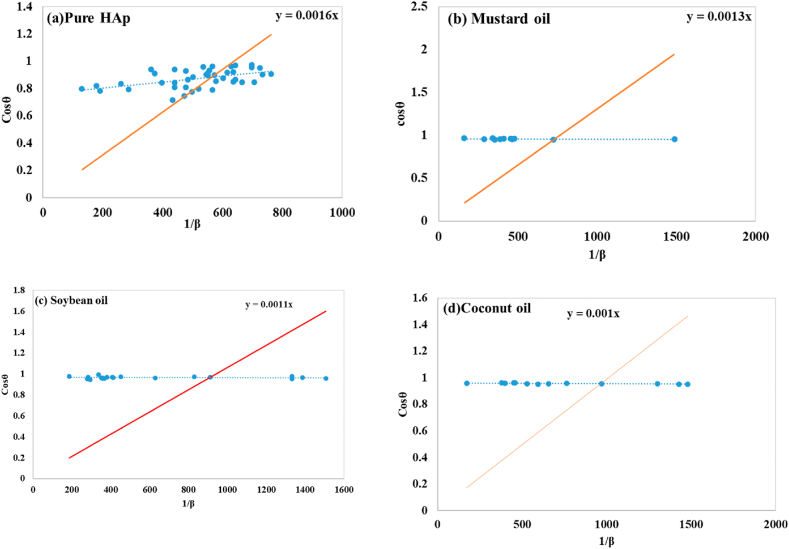
Determination of crystallite size using Sahadat-Scherre's plot for (a) Pure HAp (b) Mustard oil (c) Soybean oil, and (d) Coconut oil.

In this model 1/ β and cosθ are applied in horizontal as well as vertical axis to build a straight line across the origin (shown in Fig. 9(a–d)). An intercept model was developed using excel, matching the y = mx equation. The crystallite size was determined by comparing the slope with Kλ/D_(S_–_S)_. The model reported the magnitude of slopes as 0.0016 for pure HAp, 0.0013 for mustard oil, 0.0011 for soybean oil, and 0.0010 for coconut oil, additionally the crystal size as 86.66 nm for pure HAp, 106.66 nm for mustard oil, 126.05 nm for soybean oil, and 138.65 nm for coconut oil, correspondingly.

## 4 Conclusion

The synthesis of HAp by the wet chemical precipitation technique is further explored with the use of X-ray diffraction (XRD) analysis for their structural and crystalline characteristics. To estimate various elastic parameters for example energy density, stress, and inherent strain, several techniques e.g. Scherer's method, Stress-Strain plot method, Halder-Wagner method, and different models of Williamson-Hall such as UDM, UDEDM, and USDM commonly used. Although the value of crystallite dimension for each of the samples are almost similar except for mustard oil (6.57 nm) in the Halder- Wagner model, after comparing all these models it is unequivocally, stated that the Williamson-hall model provides the more precise crystallite size along with accurate stress, strain, as well as energy density value. Finally, the models evaluated crystallite sizes below 150 nm for the synthesized HAp nanocrystal utilizing different oil mediums, except the linear straight-line method (LSLM) that ended up resulting in a crystallite size of 693.27 nm for Pure HAp along with Soybean and Coconut oil, and 13865.4 nm for Mustard oil.

## Data availability statement

Data will be made available on request and no data was stored in any publicly available repository.

## CRediT authorship contribution statement

**Md. Kawsar:** Writing – original draft, Formal analysis, Data curation. **Md. Sahadat Hossain:** Writing – review & editing, Supervision, Methodology, Formal analysis, Conceptualization. **Newaz Mohammed Bahadur:** Supervision. **Samina Ahmed:** Supervision, Resources, Project administration, Funding acquisition.

## Declaration of competing interest

The authors declare the following financial interests/personal relationships which may be considered as potential competing interests:There is nothing to declare If there are other authors, they declare that they have no known competing financial interests or personal relationships that could have appeared to influence the work reported in this paper.

## References

[1] Hossain Md S., Mahmud M., Mobarak M.B., Sultana S., Shaikh Md A.A., Ahmed S. (Nov. 2022). New analytical models for precise calculation of crystallite size: application to synthetic hydroxyapatite and natural eggshell crystalline materials. Chem. Pap..

[2] Hamdaoui N., Azizian-Kalandaragh Y., Zaidi B., Akl A.A. (May 2021). Evolution of microstructure, strain and physical properties of quaternary nanoparticles La0.8−xCexAg0.2MnO3 perovskites. Appl. Phys. A.

[3] Materials | free full-text | X-ray diffraction analysis and Williamson-Hall method in USDM model for estimating more accurate values of stress-strain of unit cell and super cells (2 × 2 × 2) of hydroxyapatite, confirmed by ultrasonic pulse-echo test.” Accessed: August. 20, 2023. [Online]. Available: https://www.mdpi.com/1996-1944/14/11/2949. 10.3390/ma14112949 PMC8198306 34070721

[4] Polymers | free full-text | new approach for preparing in vitro bioactive scaffold consisted of Ag-doped hydroxyapatite + polyvinyltrimethoxysilane.” Accessed: August. 20, 2023. [Online]. Available: https://www.mdpi.com/2073-4360/13/11/1695. 10.3390/polym13111695 PMC8196823 34067319

[5] Characterization of nanocrystalline materials by X-ray line profile analysis | SpringerLink.” Accessed: August. 20, 2023. [Online]. Available: https://link.springer.com/article/10.1007/s10853-006-0696-1.

[6] Falsafi S.R., Rostamabadi H., Assadpour E., Jafari S.M. (Jun. 2020). Morphology and microstructural analysis of bioactive-loaded micro/nanocarriers via microscopy techniques; CLSM/SEM/TEM/AFM. Adv. Colloid Interface Sci..

[7] Hassanien A.S., Akl A.A. (Nov. 2018). X-ray studies: CO2 pulsed laser annealing effects on the crystallographic properties, microstructures and crystal defects of vacuum-deposited nanocrystalline ZnSe thin films. CrystEngComm.

[8] Rabiei M., Palevicius A., Monshi A., Nasiri S., Vilkauskas A., Janusas G. (Aug. 2020). Comparing methods for calculating nano crystal size of natural hydroxyapatite using X-ray diffraction. Nanomaterials.

[9] An analytical model for the determination of crystallite size and crystal lattice microstrain distributions in nanocrystalline materials from the variance of the X-ray diffraction peaks | SpringerLink.” Accessed: August. 20, 2023. [Online]. Available: https://link.springer.com/article/10.1007/s00339-008-4732-7.

[10] Kunka C., Boyce B.L., Foiles S.M., Dingreville R. (Nov. 2019). Revealing inconsistencies in X-ray width methods for nanomaterials. Nanoscale.

[11] Scherrer P. (1918). Estimation of the size and internal structure of colloidal particles by means of röntgen. Nachr. Ges. Wiss. Göttingen.

[12] An extensive comparative study for microstructural properties and crystal imperfections of Novel sprayed Cu3SbSe3 Nanoparticle-thin films of different thicknesses - ScienceDirect.” Accessed: August. 20, 2023. [Online]. Available: https://www.sciencedirect.com/science/article/abs/pii/S0030402620316582.

[13] Monshi A., Foroughi M.R., Monshi M.R. (2012). Modified scherrer equation to estimate more accurately nano-crystallite size using XRD. WJNSE.

[14] Monshi A., Messer P.F. (Jul. 1991). Ratio of slopes method for quantitative X-ray diffraction analysis. J. Mater. Sci..

[15] Nath D., Singh F., Das R. (Jan. 2020). X-ray diffraction analysis by Williamson-Hall, Halder-Wagner and size-strain plot methods of CdSe nanoparticles- a comparative study. Mater. Chem. Phys..

[16] X-ray analysis by Williamson-Hall and size-strain plot methods of ZnO nanoparticles with fuel variation.” Accessed: August. 20, 2023. [Online]. Available: https://www.scirp.org/html/4-4400115_43743.htm.

[17] Yousefi S., Ghasemi B., Nikolova M.P. (Sep. 2022). Morpho/Opto-structural characterizations and XRD-assisted estimation of crystallite size and strain in MgO nanoparticles by applying Williamson–Hall and size–strain techniques. J. Cluster Sci..

[18] Rattan S., Fawcett D., Poinern G.E.J., Murdoch Applied Nanotechnology Research Group (2021). Department of Physics, Energy Studies and Nanotechnology, Murdoch University, Murdoch, Western Australia 6150, Australia, “Williamson-Hall based X-ray peak profile evaluation and nano-structural characterization of rod-shaped hydroxyapatite powder for potential dental restorative procedures,”. AIMS Materials Science.

[19] Murrieta A.C., Cavazos-Cavazos D., Santos-Aguilar P., Cholula-Díaz J.L., Contreras-Torres F.F. (Jan. 2021). Microstructure of polycrystalline gold nanoparticles and thin-films from a comparative X-ray line profile analysis. Mater. Chem. Phys..

[20] Suryani S., Heryanto H., Rusdaeni R., Fahri A.N., Tahir D. (Aug. 2020). Quantitative analysis of diffraction and infra-red spectra of composite cement/BaSO4/Fe3O4 for determining correlation between attenuation coefficient, structural and optical properties. Ceram. Int..

[21] Shashidharagowda H., Mathad S. (2019). Effect of incorporation of copper on structural properties of spinel nickel manganites by co-precipitation method. Materials Science for Energy Technologies.

[22] Ghasemi Hajiabadi M., Zamanian M., Souri D. (Aug. 2019). Williamson-Hall analysis in evaluation of lattice strain and the density of lattice dislocation for nanometer scaled ZnSe and ZnSe:Cu particles. Ceram. Int..

[23] Tagliente M.A., Massaro M. (Apr. 2008). Strain-driven (002) preferred orientation of ZnO nanoparticles in ion-implanted silica. Nucl. Instrum. Methods Phys. Res. Sect. B Beam Interact. Mater. Atoms.

[24] Jacob R., Isac J. (2015). X-ray diffraction line profile analysis of Ba0.6Sr0.4FexTi(1-x) O3-δ, (x=0.4). Int. J. Chem. Stud..

[25] Mahalingam T., Vikraman D., Chandramohan R., Rhee J.-K. (Feb. 2012). Microstructural properties of electrochemically synthesized ZnSe thin films. J. Mater. Sci..

[26] Effects of annealing temperature on some structural and optical properties of ZnO nanoparticles prepared by a modified sol–gel combustion method.” Accessed: August. 21, 2023. [Online]. Available: https://www.infona.pl/resource/bwmeta1.element.elsevier-ba594c73-95c5-368a-a535-eb5957bbe4ed.

[27] Jevtić M., Mitrić M., Škapin S., Jančar B., Ignjatović N., Uskoković D. (Jul. 2008). Crystal structure of hydroxyapatite nanorods synthesized by sonochemical homogeneous precipitation. Cryst. Growth Des..

[28] Advanced materials based on nanosized hydroxyapatite - pmc.” Accessed: December. 18, 2023. [Online]. Available: https://www.ncbi.nlm.nih.gov/pmc/articles/PMC8198166/.

[29] Karampour H., Parsa M.A., Moghadam A.H., Pourhasan B., Ashiri R. (Jan. 2022). Facile solution-based synthesis of impurity-free hydroxyapatite nanocrystals at ambient conditions. J. Mater. Res. Technol..

[30] Cox S. (Jan. 2014).

[31] Hossain Md S., Shaikh Md A.A., Rahaman Md S., Ahmed S. (2022). Modification of the crystallographic parameters in a biomaterial employing a series of gamma radiation doses. Mol. Syst. Des. Eng..

[32] Bin Mobarak M. (Mar. 2022). Probing the photocatalytic competency of hydroxyapatite synthesized by solid state and wet chemical precipitation method. J. Mol. Struct..

[33] Akl A.A., Hassanien A.S. (Sep. 2015). Microstructure and crystal imperfections of nanosized CdS Se1− thermally evaporated thin films. Superlattice. Microst..

[34] A. A. Akl, S. A. Aly, and M. A. Kaid, “Microstructural and electrical properties of (WO3) 1-x (MoO3) x thin films synthesized by spray pyrolysis technique”, Accessed: November. 2, 2023. [Online]. Available: https://www.researchgate.net/profile/Safwat-Aly-3/publication/313795072_Microstructural_and_Electrical_Properties_of_WO31-xMoO3x_Thin_Films_Synthesized_by_Spray_Pyrolysis_Technique/links/58a634c2a6fdcc0e07864d27/Microstructural-and-Electrical-Properties-of-WO31-xMoO3x-Thin-Films-Synthesized-by-Spray-Pyrolysis-Technique.pdf.

[35] Vinoth Kumar K.C. (Jan. 2021). Spectral characterization of hydroxyapatite extracted from Black Sumatra and Fighting cock bone samples: a comparative analysis. Saudi J. Biol. Sci..

[36] Promotional Effect of Lanthana on the High-Temperature Thermal Stability of Pt/TiO 2 Sulfur-Resistant Diesel Oxidation Catalysts - RSC Advances (RSC Publishing) DOI:10.1039/C7RA00582B.” Accessed: August. 3, 2023. [Online]. Available: https://pubs.rsc.org/en/content/articlehtml/2017/ra/c7ra00582b.

[37] Saikiran A., Vivekanand M., Prahalad M., Yuvan S., Rameshbabu N. (2020). Microwave synthesis of Zn/Mg substituted and Zn/Mg-F co-substituted nanocrystalline hydroxyapatite. Mater. Today: Proc..

[38] Holzwarth U., Gibson N. (Sep. 2011). The Scherrer equation versus the ‘Debye-Scherrer equation. Nat. Nanotechnol..

[39] Hossain Md S., Mahmud M., Mobarak M.B., Ahmed S. (Mar. 2022). Crystallographic analysis of biphasic hydroxyapatite synthesized by different methods: an appraisal between new and existing models. Chem. Pap..

[40] Hossain Md S., Ahmed S. (2022). Synthesis of nano-crystallite gypsum and bassanite from waste *Pila globosa* shells: crystallographic characterization. RSC Adv..

[41] Microstructural study and crystallite size analysis of chemically grown bougainvillea flower-like zinc oxide nanostructures - ScienceDirect.” Accessed: August. 10, 2023. [Online]. Available: https://www.sciencedirect.com/science/article/abs/pii/S2214785322026372.

[42] Olatunde O.C., Marzouki R., Brahmia A., Onwudiwe D.C. (Jul. 2023). Lattice strain analysis of antimony sulphide nanorods. J. Cluster Sci..

[43] Hassanien A.S., Akl A.A., Sáaedi A.H. (2018). Synthesis, crystallography, microstructure, crystal defects, and morphology of Bi x Zn 1- x O nanoparticles prepared by sol–gel technique. CrystEngComm.

[44] Khorsand Zak A., Majid W. H. Abd, Abrishami M.E., Yousefi R. (Jan. 2011). X-ray analysis of ZnO nanoparticles by Williamson–Hall and size–strain plot methods. Solid State Sci..

[45] Estimation of lattice strain in ZnO nanoparticles: X-ray peak profile analysis | SpringerLink.” Accessed: August. 18, 2023. [Online]. Available: https://link.springer.com/article/10.1007/s40094-014-0141-9.

[46] Williamson-Hall analysis in estimation of lattice strain in nanometer-sized ZnO particles | SpringerLink.” Accessed: August. 18, 2023. [Online]. Available: https://link.springer.com/article/10.1186/2251-7235-6-6.

[47] Sarkar S., Das R. (2018). Shape effect on the elastic properties of Ag nanocrystals. Micro & Nano Lett..

[48] Akl A.A., El Radaf I.M., Hassanien A.S. (2020). Intensive comparative study using X-Ray diffraction for investigating microstructural parameters and crystal defects of the novel nanostructural ZnGa2S4 thin films. Superlattice. Microst..

[49] (PDF) Estimation of accurate size, lattice strain using Williamson-Hall models, SSP and TEM of Al doped ZnO nanocrystals.” Accessed: August. 19, 2023. [Online]. Available: https://www.researchgate.net/publication/330848440_Estimation_of_accurate_size_lattice_strain_using_Williamson-Hall_models_SSP_and_TEM_of_Al_doped_ZnO_nanocrystals.

[50] Mechanical properties of hydroxyapatite formed at physiological temperature | SpringerLink.” Accessed: August. 19, 2023. [Online]. Available: https://link.springer.com/article/10.1007/BF00120289.

[51] X-ray line broadening from filed aluminium and wolfram - ScienceDirect.” Accessed: August. 19, 2023. [Online]. Available: https://www.sciencedirect.com/science/article/abs/pii/0001616053900066.

[52] Nanomaterials | free full-text | strain and grain size determination of CeO2 and TiO2 nanoparticles: comparing integral breadth methods versus rietveld, μ-Raman, and TEM.” Accessed: August. 19, 2023. [Online]. Available: https://www.mdpi.com/2079-4991/11/9/2311. 10.3390/nano11092311 PMC8469540 34578630

[53] ShunmugaSundaram P., Sangeetha T., Rajakarthihan S., Vijayalaksmi R., Elangovan A., Arivazhagan G. (Jul. 2020). XRD structural studies on cobalt doped zinc oxide nanoparticles synthesized by coprecipitation method: Williamson-Hall and size-strain plot approaches. Phys. B Condens. Matter.

[54] Quantitative analysis of X-Ray diffraction spectra for determine structural properties and deformation energy of Al, Cu and Si - IOPscience.” Accessed: August. 11, 2023. [Online]. Available: https://iopscience.iop.org/article/10.1088/1742-6596/1317/1/012052.

[55] Akl A.A., Hassanien A.S. (2021). Comparative microstructural studies using different methods: effect of Cd-addition on crystallography, microstructural properties, and crystal imperfections of annealed nano-structural thin CdxZn1-xSe films. Phys. B Condens. Matter.

[56] 1988AuJPh..41..229H page 229.” Accessed: August. 19, 2023. [Online]. Available: https://adsabs.harvard.edu/full/1988AuJPh..41..229H.

[57] Halder N.C., Wagner C.N.J. (Feb. 1966). Separation of particle size and lattice strain in integral breadth measurements. Acta Crystallogr..

[58] Motevalizadeh L., Heidary Z., Ebrahimizadeh Abrishami M. (May 2014). Facile template-free hydrothermal synthesis and microstrain measurement of ZnO nanorods. Bull. Mater. Sci..

[59] Crystallographic characterization of naturally occurring aragonite and calcite phase: rietveld refinement - ScienceDirect.” Accessed: August. 19, 2023. [Online]. Available: https://www.sciencedirect.com/science/article/pii/S1319610323000534.

